# Sex and macroeconomic differences and trends in early attempts at cigarette smoking among adolescents: findings from 147 countries

**DOI:** 10.1186/s12916-022-02512-z

**Published:** 2022-09-22

**Authors:** Huaqing Liu, Qi Qi, Ying Duan, Chuanwei Ma, Chengchao Zhou

**Affiliations:** 1 Centre for Health Management and Policy Research, School of Public Health, Cheeloo College of Medicine, Shandong University, Jinan, China; 2 School of Public Health, Bengbu Medical College, Bengbu, China; 3 Department of Epidemiology, School of Public Health, Cheeloo College of Medicine, Shandong University, Jinan, China; 4 NHC Key Lab of Health Economics and Policy Research, Shandong University, 44 Wen-hua-xi Road, Jinan, 250012 Shandong China

**Keywords:** Adolescents, Age at smoking initiation, Tobacco use, Macroeconomic development

## Abstract

**Background:**

Most tobacco users initiate smoking during adolescence. Little is known about the global prevalence and trends in early cigarette smoking among adolescents. This study aimed to evaluate the prevalence of early attempts at cigarette smoking and its change trends among young adolescents.

**Methods:**

We used data from the Global Youth Tobacco Surveys on adolescents aged 12–16 years, comprising 456,634 participants from 147 countries between 2006 and 2018, to estimate the prevalence of early attempts at cigarette smoking and age distribution at attempt by sex, country income, purchasing power parity (PPP) per capita, and WHO region. We assessed the average annual rate of reduction (AARR) in the prevalence of attempts at cigarette smoking before 12 years of age in 70 countries that had data from three or more surveys completed between 1999 and 2018.

**Results:**

The average prevalence of early attempts at cigarette smoking was 12.2% (95% CI: 10.9–13.5) for boys and 6.7% (95% CI: 5.8–7.6) for girls, with the highest prevalence of 17.4% for boys and 10.7% for girls in the European region. Along with the growth of the national economy, the prevalence of early attempts at cigarette smoking gradually increased in both sexes. A total of 22.9% and 30% of countries had a negative change in AARR for boys and for girls, respectively. The countries with an upward prevalence were mainly located in the Eastern Mediterranean, Southeast Asia, and African regions. The age distribution at first cigarette smoked did not differ substantially between sexes. Notably, the age at first cigarette smoked of 10.7 years for girls was significantly earlier than that of 11.8 years for boys in low-income countries. Among cigarette-smoking adolescents, the average percentage of girls reporting smoking their first cigarette at an age <12 years was 55.7% in Q1 for PPP quintiles, 46.5% in Q2, 40.3% in Q3, 38.4% in Q4, and 34.6% in Q5, and the corresponding prevalence for boys was 46.0% in Q1, 42.8% in Q2, 42.9% in Q3, 43.5% in Q4, and 41.1% in Q5.

**Conclusions:**

The global prevalence of early attempts at cigarette smoking among adolescents was substantial, with differences by sex and macroeconomic situation, and our findings stress that interventions and policies targeting the first smoking experience are required to prevent the initiation of tobacco use among early adolescents, especially girls in low-income countries.

## Background

Tobacco use is a leading preventable risk factor for noncommunicable chronic diseases and premature death worldwide, resulting in more than eight million deaths each year and 170.9 million disability-adjusted life-years lost [1]. In 2019, more than one billion individuals worldwide were tobacco users. Therefore, preventing tobacco use is critical for tobacco control and ending the tobacco epidemic.

Most tobacco users initiate smoking during adolescence [2–4]. Hence, tobacco use is referred to as a “paediatric disease” [5, 6]. A recent study in 2019 estimated that 82.6% of tobacco users aged 20–54 years initiated smoking between ages 14 and 25, and 18.5% of tobacco users began smoking regularly by age 15 [7]. In Africa, 9.6% of adolescents in Cote d’Ivoire initiated smoking at ages 12 or 13, and 2.66% of adolescents in Mali initiated smoking at 7 years or younger [8]. In European countries, among regular smokers aged 15–39 years, the average initiation age of regular smoking was 16.6 years, and 68.1% of participants began smoking regularly before the age of 18 years [4]. Behavioral and biological studies have indicated that young individuals are particularly vulnerable to addiction and that most adult tobacco users regret starting smoking. Notably, younger individuals who initiate tobacco use are more likely to become regular and heavy tobacco users [9, 10], less likely to make successful quit attempts [11, 12], and more likely to experience tobacco-related mortality [13]. If people do not become regular tobacco users by the age of 25, they are much less likely to become tobacco users [5].

Early initiation of tobacco use during adolescence might be a critical point to control the tobacco epidemic. Tobacco addiction, as the early initiation of tobacco use, is associated with an increased risk for later nicotine dependence [14]. Therefore, preventing early tobacco-smoking initiation during adolescence could be an effective strategy to reduce the number of new tobacco users and is particularly critical in controlling tobacco use [3, 15]. However, to date, little is known about the prevalence and trends in the early initiation of cigarette use in adolescents worldwide.

In addition, the age at first tobacco use could reflect health aspects of a population. Along with the prevalence or incidence of tobacco use, the age at first smoking attempt could be regarded as a predictor of the health impact from tobacco-induced diseases, the economic impact from tobacco-attributable direct medical expenses, and lost earnings from loss of productivity [16]. Moreover, evaluation of the age at first smoking attempt could have social implications and help formulate locally relevant, targeted public health policy to reduce early initiation of tobacco use. Information on changes in early attempts at cigarette smoking may provide valuable insight into the effectiveness of adolescent-targeted prevention measures on tobacco use.

Our study was designed to evaluate the prevalence and trends in early attempts at cigarette smoking among adolescents and to further assess the age at first attempt among smoking adolescents. The results provide important insights into developing targeted intervention programs or policies to control the early initiation of tobacco use in adolescents from a public health and global perspective.

## Methods

### Study design and participants

Data were obtained from the Global Youth Tobacco Survey (GYTS), a nationally representative school-based survey of young adolescents, to collect comprehensive tobacco use and initiation information and enhance countries’ capacity to implement and evaluate tobacco prevention and control programs. The GYTS uses a consistent and standardized sample design, “core questionnaire,” and a data collection protocol to generate comparable data across countries. A two-stage sample design was used to select schools with a probability proportional to enrolment size and randomly select classes within selected schools in all countries [17]. Each student in the selected classes was eligible to participate in the GYTS. A standardized set of survey questions (i.e., core questionnaire) was used through anonymous, confidential, and self-administered surveys in all countries. The methodology of the GYTS is described in more detail on the websites of the World Health Organization (WHO) and the US Centers for Disease Control and Prevention. The country datasets are publicly available and comply with the corresponding national ethical board review.

This study included a total of 147 countries or territories that reported a nationally representative sample, after excluding surveys for which the unweighted sample of cigarette users was less than 100 or there was no data on the age at first smoking attempt. Finally, 456,634 young adolescents aged 12–16 years were analyzed. The most recent survey was used to analyze the prevalence of early attempts at cigarette smoking (i.e., trying the first cigarette before 12 years of age) if a country had conducted two or more surveys. Data from 70 countries that had three or more surveys completed between 1999 and 2018 were selected to evaluate the time trends in the prevalence of early attempts at cigarette smoking.

### Procedures

Age at first attempt at cigarette smoking was defined by the question “How old were you when you first tried a cigarette?” in the questionnaire. The responses included “I have never tried smoking a cigarette,” “7 years old or younger,” “8-9 years old,” “10-11 years old,” “12-13 years old,” “14-15 years old,” and “16 years old or older.” We calculated the mean estimates of the age at first cigarette smoked after recoding the responses as follows: “7 years old or younger” was coded as “6.5 years,” “8-9 years old” as “8.5 years,” “10-11 years old” as “10.5 years,” “12-13 years old” as “12.5 years,” “14-15 years old” as “14.5 years,” and “16 years old or older” as “16 years.” Age was defined by the question “How old are you?” The responses were “11 years old or younger,” “12 years old,” “13 years old,” “14 years old,” “15 years old,” “16 years old,” and “17 years old or older.” Our study limited the analysis to adolescents from 12 to 16 years old because these ages have a specific and clear definition in most countries. Boys and girls were included in the study. Regions were categorized into Africa, America, Southeast Asia, Europe, Eastern Mediterranean, and Western Pacific, according to the WHO [18]. Country income was categorized into four levels for analysis (i.e., low income, lower-middle income, upper-middle income, and high income) according to World Bank Analytical classifications based on the gross national income per capita for the corresponding most recent GYTS year [19]. Data on PPP per capita were obtained from the World Bank and Index Mundi [20] according to the corresponding most recent survey year of the GYTS. In this study, we classified the PPP/capita into five categories according to its integral quintiles, namely, Q1: $600–$3299, Q2: $3300–7999, Q3: $8000–13,699, Q4: $13,700–23,999, and Q5: $≥24,000. FCTC ratification status was defined based on the year that a country had ratified the WHO FCTC and the year that the GYTS survey was carried out.

### Statistical analysis

Statistical analyses were conducted using Stata v16.0. The unweighted national sample size and the number of smokers were calculated, and then the samples were weighted computed using primary sampling units, sampling weights, and strata based on the methodology of the GYTS in each country. The weighted prevalence of early attempts at cigarette smoking and the age distribution at first smoking attempt by sex were calculated for each country. We further estimated the average prevalence and 95% confidence intervals (CI) for all countries or each subgroup by country income, PPP/capita, and WHO region. The median age at first cigarette smoked for boys and girls was estimated for each country, and the mean estimate and 95% CI by country income level, PPP/capita, and WHO region were calculated. Nonoverlapping 95% CI were considered statistically significant differences according to sex, country income level, PPP/capita, and WHO region, which is a conservative estimation of the differences. Linear regression was used to assess linear trends across quintiles of the country’s PPP/capita with the mean age at first cigarette smoked, and it was also used to calculate the average annual rate of reduction (AARR) in the prevalence of early attempts at cigarette smoking before 12 years of age. The equation of AARR was created by the United Nations International Children’s Emergency Fund [21]. The age range for the first cigarette smoked was assessed using the 10th–90th percentile of the age for boys and girls.

## Results

Table 1. presents the characteristics of the study participants. A total of 456,634 adolescents aged 12–16 years from 147 countries across six WHO regions (Africa: 36; America: 27; Southeast Asia: 10; Europe: 34; Eastern Mediterranean: 20; and Western Pacific: 20) were analyzed in this study, with sample sizes ranging from 442 in the Cook Islands to 13,274 in the Russian Federation.

**Table 1:** Characteristics of Global Youth Tobacco Survey

	**Survey year**	**Number of study participants**	**Boys (%)**	**Girls (%)**	**Number of study participants initiating smoking**
**African region (n=36)**					
Algeria	2013	5164	45.8	54.2	903
Botswana	2008	1944	43.1	56.9	390
Burundi	2008	1440	45.8	54.2	192
Cabo Verde	2007	1612	45.3	54.7	180
Cameroon	2014	2306	54.5	45.5	324
Chad	2008	1471	67.0	33	163
Comoros	2007	1146	43.8	56.2	285
Congo	2009	1634	52.3	47.7	206
Côte d'Ivoire	2009	2826	55.0	45	724
Equatorial Guinea	2008	1989	47.2	52.8	277
Eritrea	2006	6896	57.7	42.3	184
Eswatini	2009	1807	43.4	56.6	174
Gabon	2014	1149	42.1	57.9	273
Gambia	2017	9339	41.4	58.6	1297
Ghana	2017	5080	47.5	52.5	294
Guinea	2008	1956	57.3	42.7	235
Kenya	2013	1666	45.5	54.5	198
Lesotho	2008	1990	39.3	60.7	221
Madagascar	2018	2112	44.0	56	476
Mali	2008	2964	53.7	46.3	622
Mauritania	2018	3065	44.9	55.1	427
Mauritius	2016	3874	46.3	53.7	1037
Mozambique	2013	4048	47.3	52.7	217
Namibia	2008	1609	43.9	56.1	294
Niger	2009	1737	48.1	51.9	169
Rwanda	2008	1322	46.1	53.9	153
Sao Tome and Principe	2010	5478	45.1	54.9	387
Senegal	2007	2227	51.0	49	287
Seychelles	2015	2313	46.7	53.3	818
Sierra Leone	2017	4440	43.3	56.7	342
South Africa	2011	5855	44.0	56	1687
Togo	2013	4135	55.5	44.5	523
Uganda	2018	2845	42.5	57.5	328
United Republic of Tanzania	2016	3465	46.6	53.4	132
Zambia	2011	2368	46.9	53.1	237
Zimbabwe	2014	5206	43.8	56.2	622
**Region of the Americas(n=27)**					
Antigua and Barbuda	2017	1934	50.4	49.6	248
Argentina	2018	1386	55.6	44.4	508
Bahamas	2013	1247	47.0	53	217
Barbados	2013	1646	50.2	49.8	339
Belize	2014	1674	43.1	56.9	382
Bolivia (Plurinational State of)	2018	4430	51.5	48.5	1091
Costa Rica	2013	2769	48.4	51.6	575
Cuba	2018	3971	49.1	50.9	864
Dominica	2009	1275	43.0	57	368
Dominican Republic	2016	1202	44.8	55.2	153
Ecuador	2016	5000	50.4	49.6	1312
El Salvador	2015	2923	47.4	52.6	846
Grenada	2016	1971	48.1	51.9	415
Guatemala	2015	3864	51.2	48.8	1265
Guyana	2015	1472	43.1	56.9	216
Honduras	2016	3281	47.4	52.6	554
Jamaica	2017	1346	44.9	55.1	433
Nicaragua	2014	3917	44.4	55.6	1158
Panama	2017	2505	49.1	50.9	245
Paraguay	2014	6365	49.8	50.2	751
Peru	2014	3420	48.2	51.8	865
Saint Lucia	2017	1442	47.5	52.5	270
Saint Vincent and the Grenadines	2011	1338	52.8	47.2	381
Suriname	2016	1749	46.8	53.2	499
Trinidad and Tobago	2017	3430	42.8	57.2	756
Uruguay	2014	4548	46.9	53.1	950
Venezuela (Bolivarian Republic of)	2010	2196	50.9	49.1	194
**Southeast Asia region(n=10)**					
Bangladesh	2013	3189	43.1	56.9	142
Bhutan	2013	1909	42.3	57.7	472
India	2009	10982	48.6	51.4	535
Indonesia	2014	5725	46.7	53.3	1464
Maldives	2011	2076	50.9	49.1	310
Myanmar	2011	2353	45.7	54.3	314
Nepal	2011	2322	47.8	52.2	205
Sri Lanka	2011	4620	45.9	54.1	286
Thailand	2015	1824	45.3	54.7	462
Timor-Leste	2013	1662	50.8	49.2	601
**European region(n=34)**					
Albania	2015	4410	47.8	52.2	1308
Armenia	2009	3161	44.4	55.6	567
Azerbaijan	2016	2139	50.1	49.9	177
Belarus	2015	2911	49.8	50.2	910
Bosnia and Herzegovina	2013	10163	50.3	49.7	3511
Bulgaria	2015	3922	47.2	52.8	1921
Croatia	2016	3173	51.4	48.6	1461
Cyprus	2011	1022	48.9	51.1	320
Czech Republic	2016	3874	49.3	50.7	1837
Estonia	2007	2946	49.2	50.8	2264
Finland	2012	4930	49.4	50.6	1949
Georgia	2017	1263	49.4	50.6	294
Greece	2013	4501	50.6	49.4	1292
Hungary	2008	3434	50.7	49.3	1960
Italy	2014	1714	52.0	48	813
Kazakhstan	2014	2023	52.8	47.2	160
Kyrgyzstan	2014	4168	44.9	55.1	731
Latvia	2014	4195	49.5	50.5	2294
Lithuania	2014	3306	48.6	51.4	1980
Malta	2017	1229	55.3	44.7	199
Montenegro	2014	3868	51.0	49	1164
North Macedonia	2016	4841	49.9	50.1	1083
Poland	2016	4922	47.8	52.2	2222
Portugal	2013	10484	46.5	53.5	2639
Republic of Moldova	2013	3794	49.3	50.7	1159
Romania	2017	5232	48.4	51.6	1302
Russian Federation	2004	13274	48.5	51.5	6595
San Marino	2014	608	49.5	50.5	166
Serbia	2017	3737	49.5	50.5	1400
Slovakia	2016	3897	49.8	50.2	1818
Slovenia	2017	2405	46.2	53.8	688
Turkey	2012	4562	51.2	48.8	1379
Ukraine	2017	3940	49.3	50.7	1097
Uzbekistan	2008	1805	43.8	56.2	151
**Eastern Mediterranean region(n=20)**					
Afghanistan	2017	1382	52.6	47.4	176
Bahrain	2015	3208	48.5	51.5	611
Djibouti	2013	1498	52.3	47.7	180
Egypt	2014	2219	58.1	41.9	331
Iran (Islamic Republic of)	2007	1597	54.8	45.2	290
Iraq	2014	1516	61.1	38.9	280
Jordan	2014	2020	54.2	45.8	504
Kuwait	2016	2319	45.3	54.7	641
Lebanon	2011	2087	47.9	52.1	521
Libya	2010	1722	50.5	49.5	177
Morocco	2016	3662	48.8	51.2	299
Oman	2016	1968	45.6	54.4	172
Pakistan	2013	7494	44.7	55.3	556
Qatar	2018	1940	49.4	50.6	373
Saudi Arabia	2010	2187	46.8	53.2	459
Sudan	2009	1420	43.2	56.8	103
Syrian Arab Republic	2010	1547	39.4	60.6	269
Tunisia	2017	2347	43.8	56.2	507
United Arab Emirates	2013	3979	44.3	55.7	888
Yemen	2014	1766	52.0	48	378
**Western Pacific region(n=20)**					
Brunei Darussalam	2013	1410	48.9	51.1	290
Cook Islands	2016	442	48.4	51.6	205
Fiji	2016	2981	44.9	55.1	537
Kiribati	2009	1263	41.6	58.4	402
Lao People's Democratic Republic	2016	5412	46.7	53.3	744
Malaysia	2009	3019	50.3	49.7	812
Marshall Islands	2016	2038	43.6	56.4	470
Micronesia (Federated States of)	2013	3286	45.8	54.2	1330
Mongolia	2014	6947	45.8	54.2	1311
New Zealand	2008	1377	59.6	40.4	481
Northern Mariana Islands	2014	2080	51.5	48.5	788
Palau	2017	1032	49.5	50.5	630
Papua New Guinea	2016	1661	48.8	51.2	540
Philippines	2015	7602	43.9	56.1	1986
Republic of Korea	2013	4059	45.5	54.5	540
Samoa	2017	1413	36.7	63.3	226
Tonga	2010	1854	43.6	56.4	739
Tuvalu	2018	588	45.1	54.9	127
Vanuatu	2017	1516	39.1	60.9	318
Viet Nam	2014	3482	48.2	51.8	283
Total	-	456 634	47.9	52.1	105 209

**Fig. 1:**
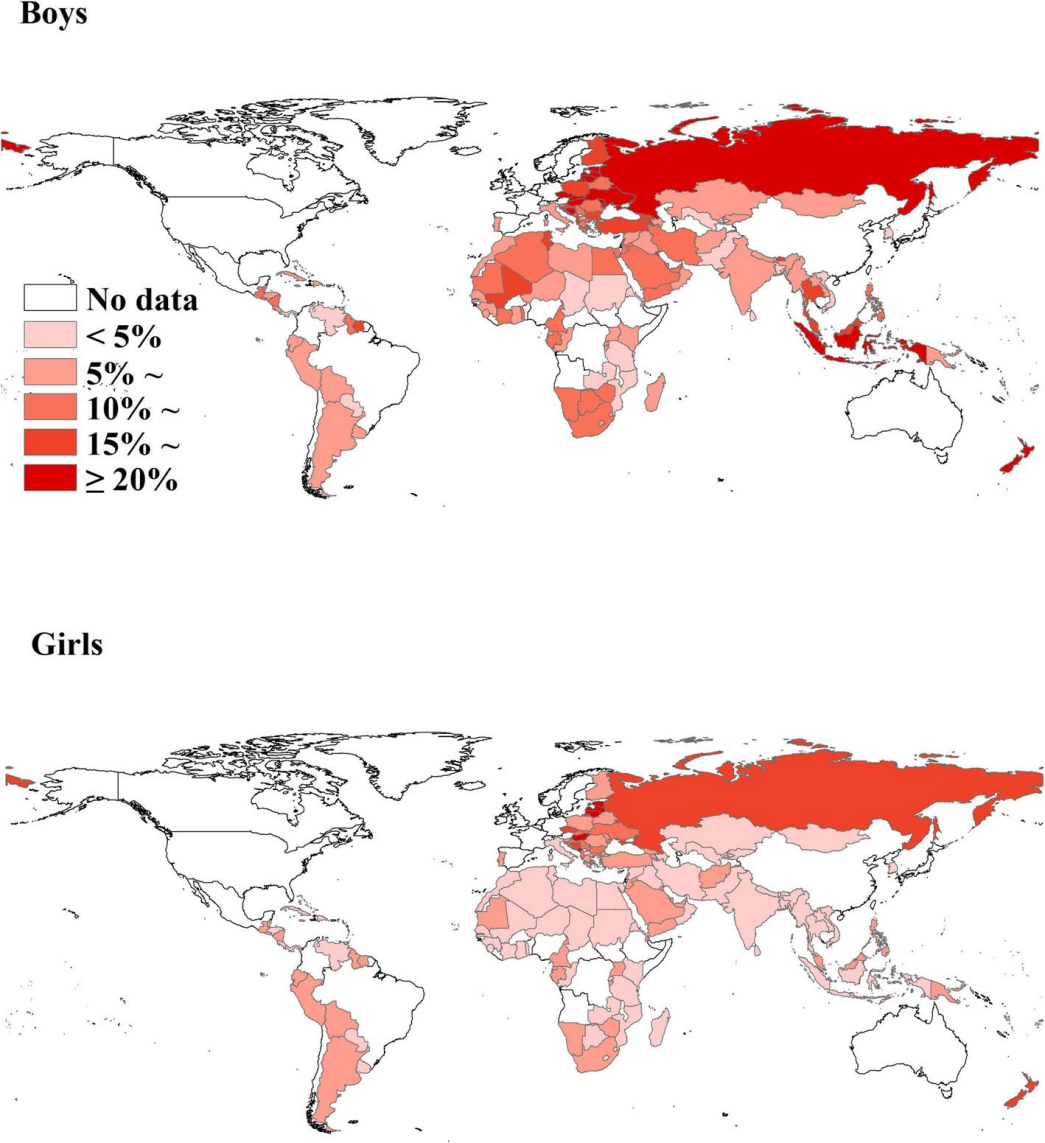
National prevalence of early attempts at cigarette smoking among adolescent boys and girls based on the most recent Global Youth Tobacco Surveys from 147 countries

Figure 1 presents the prevalence of early attempts at first cigarette smoking for boys and girls at the country level. Seventy-nine (54.1%) countries had a prevalence of early attempts at first cigarette smoking ≥ 10% for boys, with prevalence exceeding 20% in Estonia, Lithuania, the Russian Federation, Timor-Leste, Latvia, Palau, the Cook Islands, the Republic of Moldova, Bosnia and Herzegovina, Hungary, Indonesia, Micronesia, the Czech Republic, New Zealand, Slovakia, Ukraine, and Tonga, and 23 (15.8%) countries had a prevalence of early attempts at first cigarette smoking ≥ 10% for girls, with prevalence exceeding 20% in Estonia, Lithuania, the Cook Islands, Latvia, Palau, Montenegro, and Hungary. There was a high prevalence of early attempts at first cigarette smoking for girls relative to boys in Montenegro, Maldives, and Antigua and Barbuda.

Table 2 shows the prevalence of early attempts at first cigarette smoking among young adolescents according to the WHO regions, income groups, PPP/capita, and FCTC ratification status. The mean prevalence of early attempts at first cigarette smoking was 12.2% (95% CI: 10.9–13.5) for boys and 6.7% (95% CI: 5.8–7.6) for girls, with a significant difference. The difference applied widely to most WHO regions, income groups, and PPP/capita. There were variations in the prevalence of early attempts at first cigarette smoking across WHO regions, with the highest prevalence of 17.4% for boys and 10.7% for girls in the European region, which was higher than that in Africa, the Eastern Mediterranean region, and the Americas (9.1%, 9.7%, and 10.8% for boys and 4.3%, 6.3%, and 4.6% for girls, respectively). The prevalence of early attempts at cigarette smoking was 8.0% in low-income countries, 11.5% in lower-middle-income countries, 12.0% in upper-middle-income countries, and 15.6% in high-income countries for boys, and the corresponding prevalence for girls was 3.4%, 5.2%, 7.0%, and 10.1%, respectively. With increasing PPP/capita, the prevalence of early attempts at cigarette smoking increased from Q1 to Q4 but decreased in the highest quintile (Q5) for both sexes. The highest prevalence was observed in the Q4 group in terms of PPP/capita. The prevalence for boys was higher than that for girls in the other PPP/capita categories (i.e., Q1, Q2, Q3, and Q5), except for the Q4 group of PPP/capita, in which no significant sex difference was observed. However, there was no significant difference in the prevalence of early attempts at cigarette smoking by FCTC ratification status.

**Table 2:** Prevalence of early attempts at first cigarette smoking among adolescents

	***Number of countries***	***Boys***	***Girls***
***Total***	147	12.2 (10.9–13.5)	6.7 (5.8–7.6)
***WHO region***			
African region	36	9.1 (7.9–10.3)	4.3 (3.6–5.0)
Region of the Americas	27	9.7 (8.2–11.2)	6.3 (5.4–7.3)
Southeast Asia region	10	12.2 (5.1–19.3)	4.0 (1.8–6.2)
European region	34	17.3 (13.6–21.0)	10.5 (7.8–13.2)
Eastern Mediterranean region	20	10.8 (8.9–12.6)	4.6 (3.8–5.5)
Western Pacific region	20	13.6 (9.7–17.5)	8.5 (5.1–11.9)
***PPP per capita, $***			
Q1 600–3299	30	9.5 (6.9–12.2)	4.2 (3.1–5.2)
Q2 3300–7999	29	9.1 (7.5–10.7)	4.6 (3.5–5.7)
Q3 8000–13,699	29	14.0 (11.0–17.0)	7.2 (5.5–9.0)
Q4 13,700–23,999	29	15.4 (11.4–19.4)	10.1 (6.9–13.3)
Q5 ≥24,000	30	12.8 (10.2–15.5)	7.4 (5.4–9.4)
***World Bank income group***			
Low income	23	8.0 (6.4–9.6)	3.4 (2.6–4.1)
Lower-middle income	42	11.5 (9.3–13.7)	5.2 (4.2–6.2)
Upper-middle income	47	12.0 (10.2–13.7)	7.0 (5.7–8.2)
High income	34	15.5 (11.8–19.2)	9.9 (7.2–12.6)
***FCTC ratification status***			
Not ratified	10	12.2 (5.0–19.5)	5.9 (2.0–9.8)
Ratified	137	12.2 (10.9–13.5)	6.8 (5.8–7.7)

*PPP* purchasing power parity

The mean age at first cigarette smoked was 12.0 years (95% CI: 11.9–12.2) for boys and 11.9 years (95% CI: 11.6–12.1) for girls. The age window at first cigarette smoked was between 10.5 and 12.5 years for both genders (Table 3). In low-income countries, the age at first cigarette smoked among girls, 10.7 years old (95% CI: 10.0–11.4), was significantly earlier than that for boys, 11.8 years old (95% CI: 11.4–12.2). Additionally, the age at first cigarette smoked was earlier for girls in low-income countries than for girls in upper-middle-income and high-income countries, with 12.3 years old (95% CI: 12.0–12.6) and 12.4 years old (95% CI: 12.2–12.7), respectively. Girls tried smoking for the first time at a younger age than boys in the lower PPP quintiles (Q1). However, no significant sex differences were observed in the other PPP/capita categories (Q2–Q5). With increasing PPP/capita, the age at first cigarette smoked increased gradually in girls (*p* for trend < 0.001) but not in boys (*p* for trend = 0.334) (Table 3). Girls tried cigarette smoking 0.6 years, 0.6 years, and 1.0 years earlier than boys in the African, Eastern Mediterranean, and Southeast Asian regions, respectively, and the corresponding age window at first cigarette smoked was reduced to 8.5–12.5 years from 10.5 to 12.5 years.

**Table 3:** Trends in early attempts at cigarette smoking among adolescents

	Mean age at first cigarette smoked/years		Age window at first cigarette smoked (10th–90th percentile; years)	
	Boys^a^	Girls^a^	Boys	Girls
***Total***	12.0 (11.9–12.2)	11.9 (11.6–12.1)	10.5–12.5	10.5–12.5
***WHO region***				
African region	11.7 (11.4–12.1)	11.1 (10.5–11.6)	10.5–12.5	8.5–12.5
Region of the Americas	12.1 (11.8–12.4)	12.2 (11.8–12.6)	10.5–12.5	10.5–12.5
Southeast Asia region	11.9 (11.2–12.6)	10.9 (9.8–12.0)	10.5–12.5	8.5–12.5
European region	11.8 (11.5–12.2)	12.4 (12.0–12.8)	10.5–12.5	10.5–13.7
Eastern Mediterranean region	12.2 (11.9–12.5)	11.6 (10.8–12.4)	10.5–12.5	8.7–12.5
Western Pacific region	12.5 (12.0–13.0)	12.7 (12.2–13.2)	10.5–14.5	10.7–14.5
***Country income***				
Low income	11.8 (11.4–12.2)	10.7 (10.0–11.4)	10.5–12.5	8.5–12.5
Lower-middle income	12.0 (11.6–12.3)	11.7 (11.2–12.2)	10.5–12.5	10.5–12.5
Upper-middle income	12.2 (12.0–12.5)	12.3 (12.0–12.6)	10.5–12.5	10.5–12.5
High income	12.0 (11.9–12.2)	12.4 (12.2–12.7)	10.5–12.5	11.3–12.5
***PPP/capita, $***				
Q1 600–3299	11.8 (11.4–12.2)	10.8 (10.2–11.4)	10.5–12.5	8.5–12.5
Q2 3300–7999	12.2 (11.9–12.6)	11.8 (11.1–12.5)	10.5–12.5	10.5–14.5
Q3 8000–13,699	11.9 (11.4–12.3)	12.1 (11.7–12.5)	10.5–12.5	10.5–12.5
Q4 13,700–23,999	12.1 (11.8–12.4)	12.1 (11.7–12.5)	10.5–12.5	10.5–12.5
Q5 ≥24,000	12.1 (11.8–12.4)	12.5 (12.2–12.8)	10.5–12.5	12.5–12.5
*β* (95% CI)	0.05 (−0.06, 0.16)	0.36 (0.21, 0.52)	-	-
*P*	0.334	<0.001	-	-
***FCTC ratification status***				
Not ratified	12.3 (11.8–12.8)	11.7 (10.3–13.1)	10.7–12.5	8.5–14.3
Ratified	12.0 (11.8–12.2)	11.9 (11.6–12.1)	10.5–12.5	10.5–12.5

Linear regression was used to assess the linear trend across quintiles of country’s PPP/capita with mean age at first cigarette smoked

*PPP* purchasing power parity, *β* linear regression coefficient

^a^Data are mean (95% CI)

**Fig. 2:**
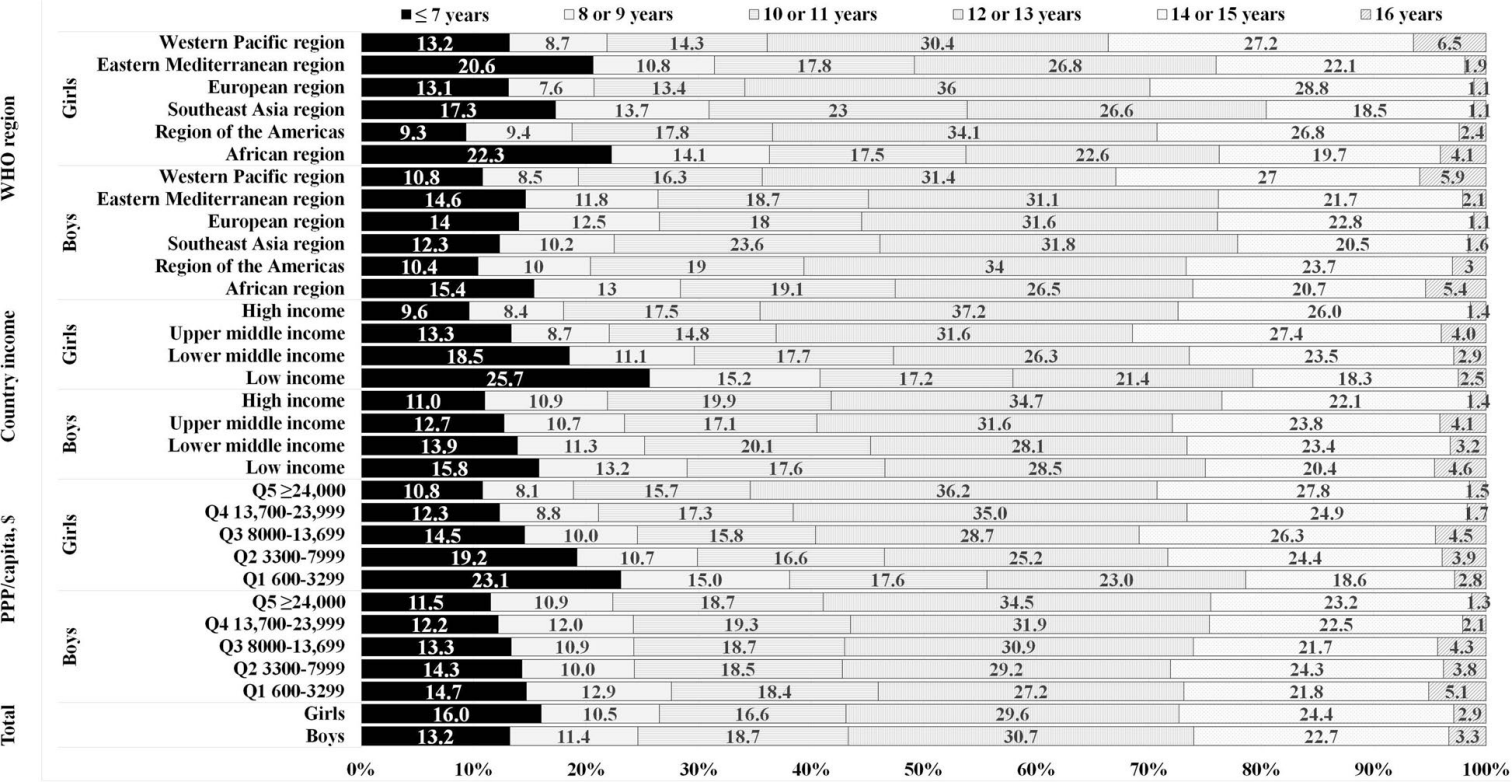
Age distribution at first cigarette smoked among adolescents according to country income, PPP, and WHO region

Figure 2 shows the age distribution at first cigarette smoked among smoking adolescent boys and girls according to country income, PPP, and WHO region. Of cigarette-smoking adolescents, 16.0% reported trying cigarette smoking at age ≤7 years, 10.5% at 8–9 years, 16.6% at 10–11 years, 29.6% at 12–13 years, 24.4% at 14–15 years, and 2.9% at 16 years for girls, and 13.2%, 11.4%, 18.7%, 30.7%, 22.7%, and 3.3% for boys, respectively. For girls, the average percentage reporting smoking their first cigarette at an age <12 years was 58.1% in low-income countries, 47.3% in lower-middle-income countries, 36.8% in upper-middle-income countries, and 35.5% in high-income countries. For PPP quintiles, the corresponding percentages were 55.7% in Q1, 46.5% in Q2, 40.3% in Q3, 38.4% in Q4, and 34.6% in Q5. For boys, the average percentage reporting smoking their first cigarette at an age <12 years was 46.6% in low-income countries, 45.3% in lower-middle-income countries, 40.5% in upper-middle-income countries, and 41.8% in high-income countries. For PPP quintiles, the average percentage was 46.0% in Q1, 42.8% in Q2, 42.9% in Q3, 43.5% in Q4, and 41.1% in Q5. More than 30% of smoking adolescents reported smoking their first cigarette at an age <12 years in all six regions, and one in two young girls tried smoking a cigarette before 12 years of age in the African region (53.9%) and Southeast Asian region (54.0%).

**Fig. 3:**
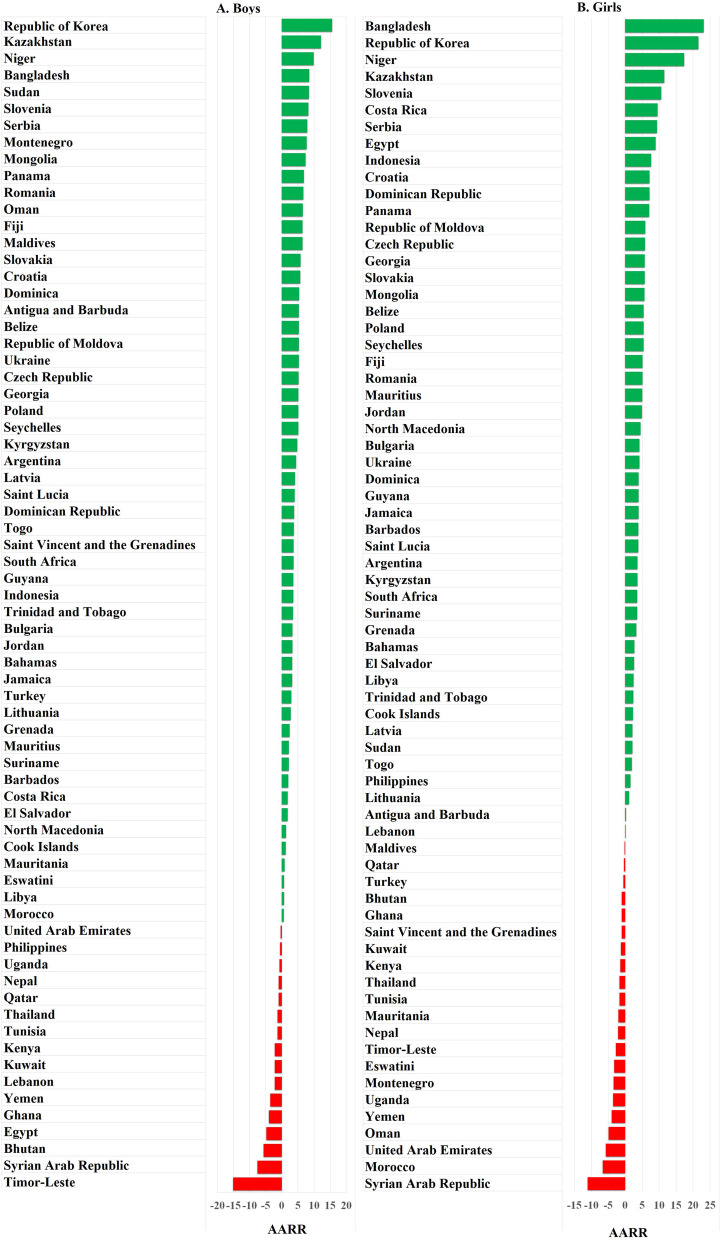
Average annual rate of reduction in the prevalence of early attempts at cigarette smoking (i.e., smoking the first cigarette before 12 years of age)

Figure 3 shows the changes in AARR in the prevalence of first cigarette smoked before 12 years of age at the national level. For boys, 77.1% (54/70) of countries had a positive change in AARR prevalence, ranging from 0.6% in Morocco to 15.6% in the Republic of Korea, while 22.9% (16/70) had a negative change in AARR, ranging from −0.4% in the United Arab Emirates to −15.1% in Timor-Leste, among which 50% (8/16) came from the Eastern Mediterranean region, 25% (4/16) came from the Southeast Asia region and 18.8% (3/16) came from the African region. For girls, 70% (49/70) of countries had a positive change in AARR in the prevalence of attempts at cigarette smoking before 12 years of age, ranging from 0.1% in Lebanon to 23.1% in Bangladesh, while 30% of countries (21/70) had a negative change in AARR, ranging from −0.2% in the Maldives to −11.1% in the Syrian Arab Republic, among which 38.1% (8/21) came from the Eastern Mediterranean region, 23.8% (5/21) came from the Southeast Asia region, and 23.8% (5/21) came from the African region.

## Discussion

Our study estimated the prevalence of early attempts at cigarette smoking among adolescents aged 12–16 years in 146 countries. One in eight adolescent boys and one in fifteen girls had smoked their first cigarette before they were 12 years old. Our findings indicated that the global prevalence of early attempts at cigarette smoking among adolescents was substantial. This is similar to the findings of a recent study [22] on the prevalence of cigarette smoking, which was 11.3% in boys and 6.1% in girls, and the prevalence in boys was approximately twice as high as that in girls. Moreover, one-quarter of smoking adolescents tried their first cigarette before 12 years of age. As matters stand, most tobacco users begin smoking during adolescence [2–4, 8], and smoking adolescents begin smoking at an earlier age. Tobacco users with higher nicotine dependence begin smoking earlier in life [23]. Moreover, age at first cigarette smoking experience is significantly associated with smoking status in the future [24, 25]. Notably, a 1-year delay in first smoking experience results in a 25% reduction in the probability of future smoking among adolescents [26]. The evidence highlights the vital importance and unique opportunity to prevent the initiation of tobacco use in early adolescence.

In this study, the prevalence was high in all six WHO regions, which is similar to the prevalence of cigarette smoking among adolescents [22], indicating that the prevention of early smoking attempts should be strengthened throughout all regions, especially in the European region, because the highest prevalence in early cigarette smoking was found for both sexes in the region. Although the prevalence of first cigarette smoked before 12 years of age declined over time in most countries, 1/3 of the countries, which are mainly located in the Eastern Mediterranean, Southeast Asia, and African regions, experienced an increase, especially for girls. Our findings also strengthen the necessity of continued and intensive actions to further control the early initiation of tobacco use in young adolescents, especially in these regions.

Our study indicated that the age distribution at first cigarette smoked did not differ substantially between sexes, although there was a sex difference in cigarette use among adolescents [22]. Conversely, young girls tended to try cigarette smoking earlier in the African, Southeast Asia, and Eastern Mediterranean regions, and the corresponding age window at first cigarette smoked was 2 years earlier than that in other regions. This result is consistent with the change in the AARR of early attempts at cigarette smoking, and the increase was mainly found in these regions. This evidence indicates an urgent need for policies and intervention programs targeting young girls in these regions. Tobacco use varies by sex and region [27] and is perhaps related to racial and social culture [28–30]. Further studies are needed to explore cultural and social mores that may prevent early attempts at cigarette smoking among young girls in these regions and help address risk factors for tobacco use initiation in early adolescence.

The fact that most smoking adolescents aged 12–16 years tried smoking a cigarette during the age window between 10 and 13 years old, assuming that 10–11 years old means 10.5 and 12–13 means 12.5 in this study, indicates that this age period is the crucial window during which young adolescents develop into tobacco users. This finding stresses that protecting adolescents from exposure to smoking during this crucial age window may be vital to controlling tobacco use among young adolescents. Most countries set their legal purchase age for tobacco at 16 or 18 years globally, which is an extensively adopted national policy curbing the initiation of tobacco use among children and adolescents. However, it is worth considering that there was still an abundance of young adolescents who smoked cigarettes. Moreover, our study did not observe that ratifying the WHO FCTC influenced early attempts at cigarette smoking among young adolescents. FCTC ratification status may not reflect the actual implementation and enforcement of the regulatory measures at the country level. There is still no common metric for publicly assessing FCTC implementation, which needs more attention in future studies. More studies are needed to investigate how tobacco products can be accessed or exposed in early adolescence. Many smoking adolescents or youths obtained their first cigarette from peers in China [31, 32] and the Czech Republic [33]. Increasing the minimum age of purchase of tobacco products may support the decrease in the number of smoking young adults [34, 35], who are associated with increased risk of early adolescents’ access or exposure to tobacco use as peers [36–38].

To our knowledge, this study is the first to investigate the relationship between national economic development and early attempts at cigarette smoking among adolescents. With the increase in national income level, the prevalence of early attempts at cigarette smoking gradually increased in both sexes. The association also applies to PPP/capita, and the prevalence gradually increased from Q1 to Q4 but decreased for Q5. With the development of the economy, the supply of cigarette products in a country increases. A recent study reported that national income levels were associated with the prevalence of cigarette use in adolescents [22]. As an index reflecting individuals’ tobacco purchasing power and national economic capacity for tobacco control, national macroeconomic development could be associated with the early initiation of tobacco use. Moreover, the risk of adolescents’ tobacco-smoking initiation is differentially affected by individuals’ disposable income [39]. Exposure to high cigarette prices is related to reducing the initiation of cigarette smoking among youth [40, 41]. At the individual level, the economy is an important factor influencing the early initiation of tobacco use.

It is remarkable that among smoking adolescents, young girls tried cigarette smoking earlier than young boys in low-income countries and lower PPP/capita categories. Our findings indicate that the age at first cigarette smoked among young girls is closely related to the national macroeconomy and purchasing power. Young girls are a socially vulnerable group who try smoking cigarettes in low-income countries. This suggests a need to raise awareness about early attempts at cigarette smoking and the need for developing intervention programs to reduce the early initiation of tobacco use in girls from a low level of national economic development. Unfortunately, the prevalence of cigarette smoking has increased along with increasing country income among girls aged 13–15 years [22] and women aged 15–49 years [42]. This indicates major issues in reducing the high prevalence of cigarette smoking in high-income countries and highlights how to prevent the early initiation of cigarette smoking in low-income countries. Decreasing the affordability of tobacco products is one of the most effective measures for preventing early initiation of tobacco use, especially among young individuals, because they are particularly sensitive to price changes [41, 43]. Interestingly, young women are more price-responsive to cigarette smoking initiation, but young men are more price-sensitive to cigarette prevalence and consumption [41]. Therefore, accompanied by the development of the national economy, expanding and strengthening fiscal policy to reduce the affordability of tobacco products is an essential component to prevent young adolescents, especially girls, from starting to smoke cigarettes.

Our study has several limitations. First, cigarette smoking and age at first cigarette smoked were self-rated via one question, and the response was categorical data, not continuous variables, such as specific age, which might be inclined to some recall or response bias. In addition, this study estimated only the age at first cigarette smoked and did not include other tobacco products, which comprise an increasing proportion of tobacco use among young adolescents [22], although smoking cigarettes is the most common form of tobacco use worldwide. Second, the GYTS on the age at first cigarette smoked among young adolescents are conducted in schools; therefore, our results might not apply to all adolescents. Moreover, as smoking is often associated with socioeconomic status, adolescents from lower socioeconomic status are likely to start smoking at a younger age and are less likely to have a school education. In light of this, the results presented in this study might underestimate age at first cigarette smoked, particularly in low-income countries or countries without free education. Third, the GYTS is a cross-sectional survey; therefore, causal inferences cannot be established. Furthermore, the study did not evaluate confounders. Further studies are needed to assess the association between exposure to environmental tobacco use, access to tobacco products, tobacco advertisements, and the initiation of cigarette smoking.

## Conclusions

Cigarette smoking often begins with the first cigarette smoked and repeated experimentation before adolescents become regular smokers. It is important to target the early stage to prevent smoking. Our findings highlight the need to adopt continued and intensive actions to reduce early attempts at cigarette smoking in young adolescents; the age range of 10–13 years is a critical age window to change the tobacco epidemic among adolescents. Young girls tend to try smoking cigarettes earlier in low-income countries, such as those in Africa, Southeast Asia, and the Eastern Mediterranean.

## Data Availability

The datasets analyzed for our study from the Global Youth Tobacco Surveys are available at the official website (https://www.who.int/teams/noncommunicable-diseases/surveillance/data).

## Abbreviations

AARR: Average annual rate of reduction
CI: Confidence intervals
FCTC: Framework Convention on Tobacco Control
GYTS: Global Youth Tobacco Surveys
PPP: Purchasing power parity
WHO: World Health Organization
